# Supplementary material for: From sequence to enzyme mechanism using multi-label machine learning
Source: BMC Bioinformatics. 2014 May 19;15:150. doi: 10.1186/1471-2105-15-150 (PMC4229970; doi:10.1186/1471-2105-15-150)
Supplement: Additional file 2 — Java code of ml2db. Additional file ml2db_code.tar.gz contains the Java source code to run the multi-label machine learning experiments and save the results to database. The code’s Javadoc is included. [file 1471-2105-15-150-S2.zip › additional file 2/ml2db/ml2db/doc/main/ml2db/weka/DataSetDbLoader.html]

DataSetDbLoader


JavaScript is disabled on your browser.


- Overview
- Package
- Class
- Use
- Tree
- Deprecated
- Index
- Help

- Prev Class
- Next Class

- Frames
- No Frames

- All Classes

- Summary:
- Nested |
- Field |
- Constr |
- Method

- Detail:
- Field |
- Constr |
- Method


main.ml2db.weka

## Class DataSetDbLoader

- java.lang.Object
- - main.ml2db.weka.DataSetDbLoader

- Direct Known Subclasses:
  :   MulanDataSetDbLoader

  ---

    

  ```
  public class DataSetDbLoader
  extends java.lang.Object
  ```

  Loads data (instances, attribute values, class values) from database using
  the queries in the data Properties.

  Version:
  :   28 Apr 2010

  Author:
  :   Luna De Ferrari ldf(at)st-andrews.ac.uk

- - ### Constructor Summary

    Constructors

    Constructor and Description

    `DataSetDbLoader(DataSetManager manager)`
  - ### Method Summary

    Methods

    | Modifier and Type | Method and Description |
    | --- | --- |
    | `java.util.Properties` | `getDataProperties()` |
    | `DataSetManager` | `getDataSetManager()` |
    | `InstAttValueMap` | `getInstanceAttributeData()` |
    | `InstAttValueMap` | `getInstanceClassData()` |
    | `InstAttValueMap` | `getMap(java.lang.String queryProperty, java.lang.String feature_or_class)` |
    | `void` | `loadData()` Create a data set and load it with data from database |

    - ### Methods inherited from class java.lang.Object

      `equals, getClass, hashCode, notify, notifyAll, toString, wait, wait, wait`

- - ### Constructor Detail


    - #### DataSetDbLoader

      ```
      public DataSetDbLoader(DataSetManager manager)
      ```
  - ### Method Detail


    - #### getDataProperties

      ```
      public java.util.Properties getDataProperties()
      ```


    - #### getDataSetManager

      ```
      public DataSetManager getDataSetManager()
      ```


    - #### getInstanceAttributeData

      ```
      public InstAttValueMap getInstanceAttributeData()
      ```


    - #### getInstanceClassData

      ```
      public InstAttValueMap getInstanceClassData()
      ```


    - #### getMap

      ```
      public InstAttValueMap getMap(java.lang.String queryProperty,
                           java.lang.String feature_or_class)
      ```


    - #### loadData

      ```
      public void loadData()
      ```

      Create a data set and load it with data from database


- Overview
- Package
- Class
- Use
- Tree
- Deprecated
- Index
- Help

- Prev Class
- Next Class

- Frames
- No Frames

- All Classes

- Summary:
- Nested |
- Field |
- Constr |
- Method

- Detail:
- Field |
- Constr |
- Method
